# Supplementary material for: Immunothrombotic Mechanisms Induced by Ingenol Mebutate Lead to Rapid Necrosis and Clearance of Anogenital Warts
Source: Int J Mol Sci. 2022 Nov 2;23(21):13377. doi: 10.3390/ijms232113377 (PMC9656782; doi:10.3390/ijms232113377)
Supplement: Supplementary file 1 [file ijms-23-13377-s001.zip › ijms-1899422-supplementary figures.pdf]

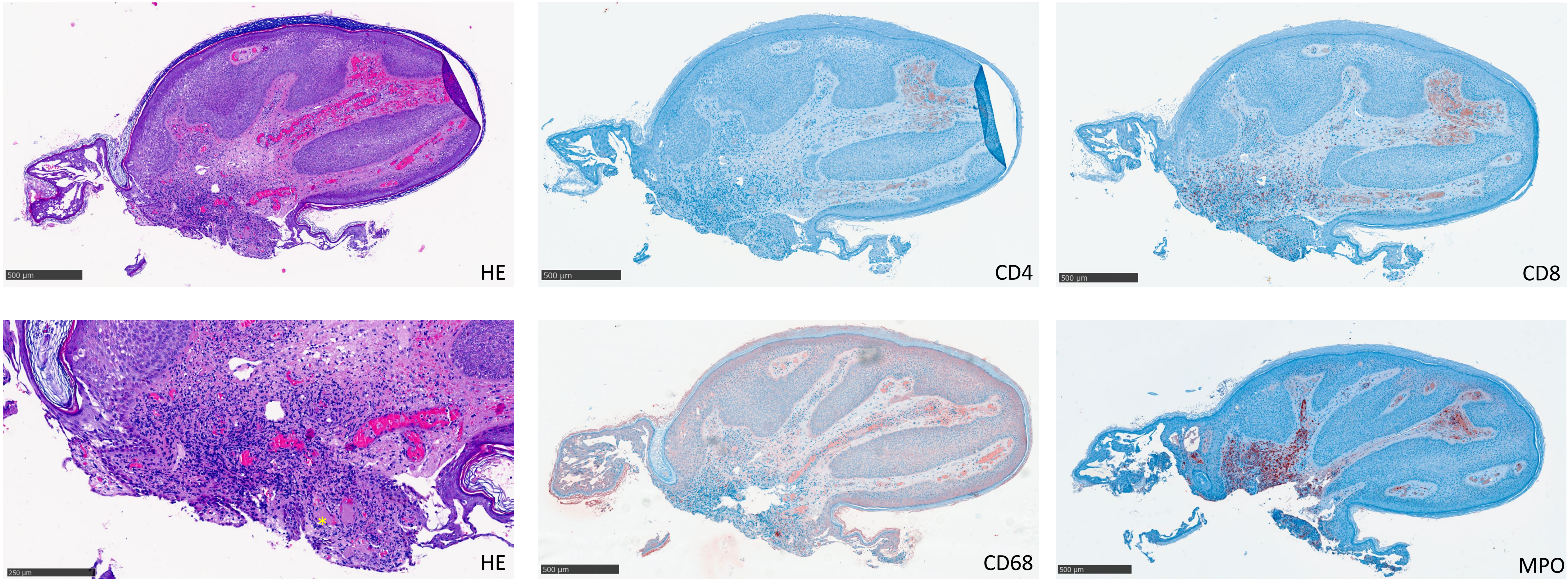

Figure S1: Histology of patient 3 after 8 hours. The HE stains showed fibrinoid thrombi in small vessels (yellow star). Immunhistochemically the inflammatory infitrat was mixed with dominance of neutrophils.

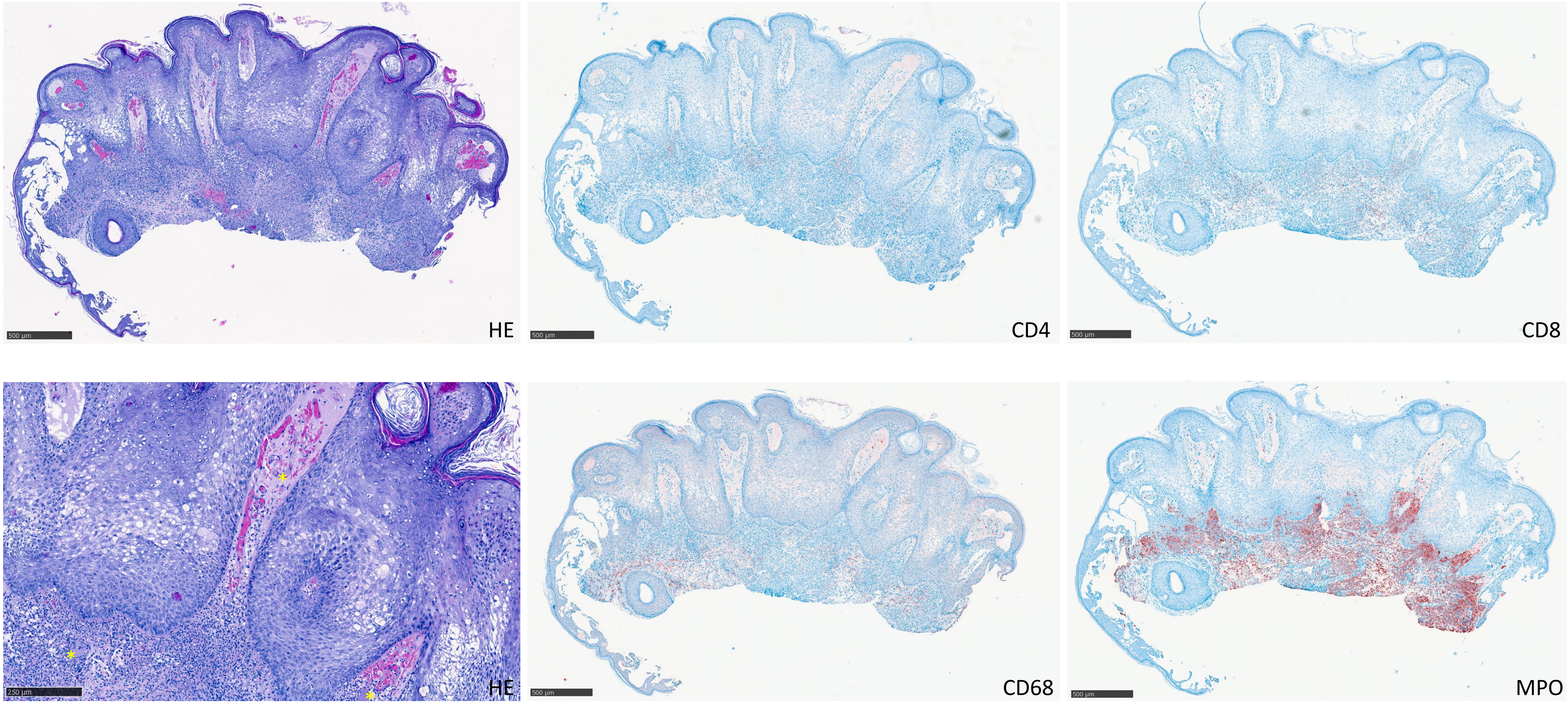

Figure S2: Histology of patient 7 after 8 hours. The HE stains showed fibrinoid thrombi in small vessels (yellow star). Immunhistochemically the inflammatory infitrat was mixed with dominance of neutrophils.

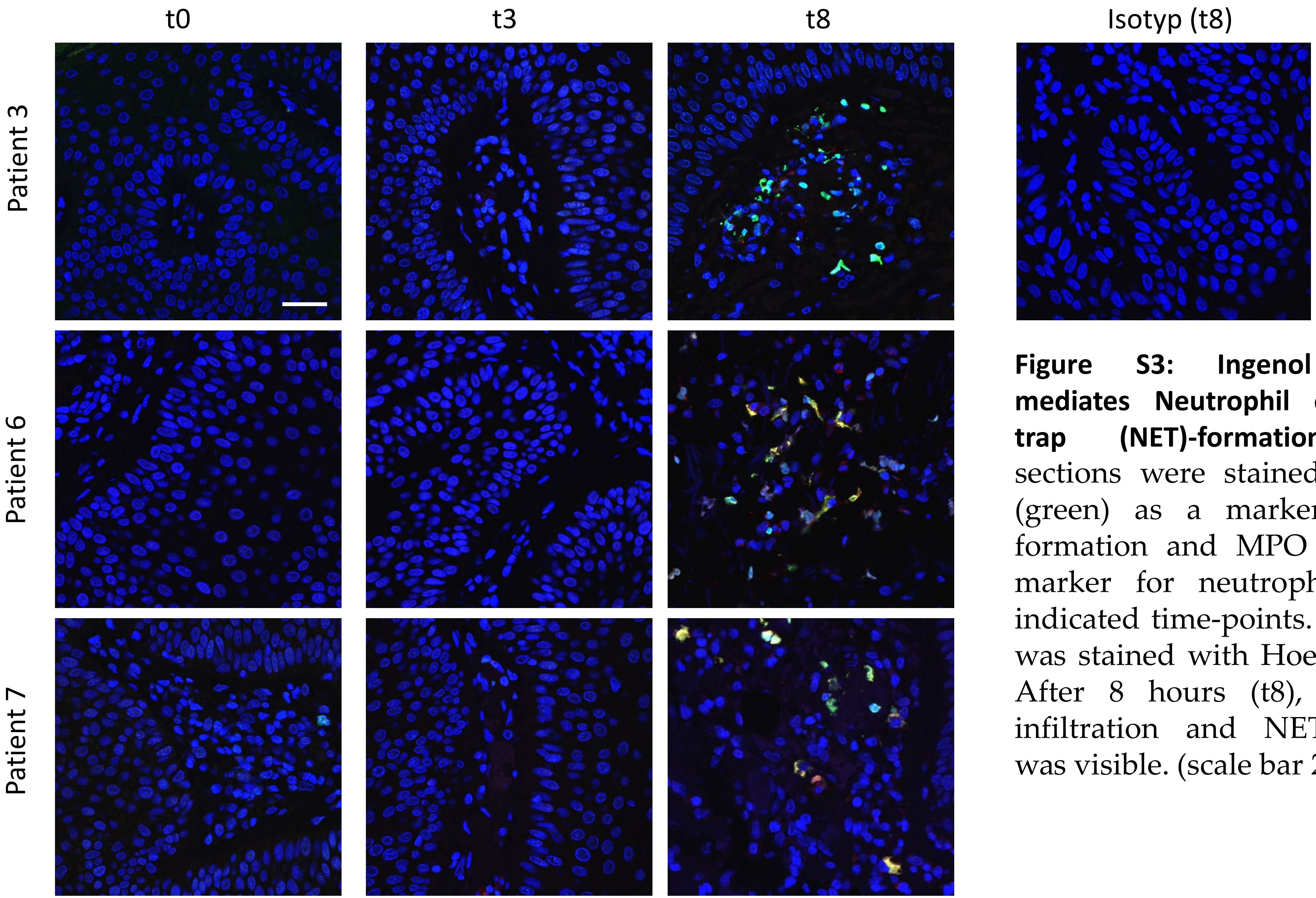

Figure S3: Ingenol mebutate mediates Neutrophil extracellular trap (NET)-formation. Tissue sections were stained for H3cit (green) as a marker of NET-formation and MPO (red) as a marker for neutrophils at the indicated time-points. Chromatin was stained with Hoechst (blue). After 8 hours (t8), neutrophil infiltration and NET-formation was visible. (scale bar 25 µm)
